# Expression and Function of ABC Proteins in Fish Intestine

**DOI:** 10.3389/fphys.2021.791834

**Published:** 2021-12-09

**Authors:** Flavia Bieczynski, Julio C. Painefilú, Andrés Venturino, Carlos M. Luquet

**Affiliations:** ^1^Centro de Investigaciones en Toxicología Ambiental y Agrobiotecnología del Comahue – Consejo Nacional de Investigaciones Científicas y Técnicas, Universidad Nacional del Comahue, Neuquén, Argentina; ^2^Instituto Patagónico de Tecnologías Biológicas y Geoambientales, Consejo Nacional de Investigaciones Científicas y Técnicas – Universidad Nacional del Comahue, Bariloche, Argentina; ^3^Laboratorio de Ecotoxicología Acuática, Subsede INIBIOMA-CEAN (CONICET – UNCo), Junín de los Andes, Argentina

**Keywords:** multixenobiotic resistance, epithelial physiology, aquatic pollutants, polarized transport, detoxification

## Abstract

In fish, the intestine is fundamental for digestion, nutrient absorption, and other functions like osmoregulation, acid-base balance, and excretion of some metabolic products. These functions require a large exchange surface area, which, in turn, favors the absorption of natural and anthropogenic foreign substances (xenobiotics) either dissolved in water or contained in the food. According to their chemical nature, nutrients, ions, and water may cross the intestine epithelium cells’ apical and basolateral membranes by passive diffusion or through a wide array of transport proteins and also through endocytosis and exocytosis. In the same way, xenobiotics can cross this barrier by passive diffusion or taking advantage of proteins that transport physiological substrates. The entry of toxic substances is counterbalanced by an active efflux transport mediated by diverse membrane proteins, including the ATP binding cassette (ABC) proteins. Recent advances in structure, molecular properties, and functional studies have shed light on the importance of these proteins in cellular and organismal homeostasis. There is abundant literature on mammalian ABC proteins, while the studies on ABC functions in fish have mainly focused on the liver and, to a minor degree, on the kidney and other organs. Despite their critical importance in normal physiology and as a barrier to prevent xenobiotics incorporation, fish intestine’s ABC transporters have received much less attention. All the ABC subfamilies are present in the fish intestine, although their functionality is still scarcely studied. For example, there are few studies of ABC-mediated transport made with polarized intestinal preparations. Thus, only a few works discriminate apical from basolateral transport activity. We briefly describe the main functions of each ABC subfamily reported for mammals and other fish organs to help understand their roles in the fish intestine. Our study considers immunohistochemical, histological, biochemical, molecular, physiological, and toxicological aspects of fish intestinal ABC proteins. We focus on the most extensively studied fish ABC proteins (subfamilies ABCB, ABCC, and ABCG), considering their apical or basolateral location and distribution along the intestine. We also discuss the implication of fish intestinal ABC proteins in the transport of physiological substrates and aquatic pollutants, such as pesticides, cyanotoxins, metals, hydrocarbons, and pharmaceutical products.

## Introduction

Aquatic animals take up food and water together; therefore, their digestive systems are exposed to xenobiotics present in the food or dissolved in water. This risk is particularly enhanced in marine teleost fish, which drink high volumes of water for osmoregulation purposes. The intestinal epithelium (mucosa) is the main surface of alkaline digestion and nutrient absorption and reabsorption of bile acids. These functions require a large surface area of epithelial tissue with a broad spectrum of inwardly directed membrane transporters. Additionally, in marine teleosts, the intestinal mucosa participates in osmoregulation and ionic and acid-base balance. Thus, the intestinal mucosa offers a large surface area for the entry of xenobiotics, either through simple diffusion or facilitated by a great diversity of proteins that primarily transport nutrients, bile acids, ions, or water. The biotransformation and active excretion of xenobiotics at the luminal membrane of the intestine mucosa limits their incorporation. Alternatively, the parent or biotransformed substance is transported by the blood to the liver and the kidney to be metabolized and excreted (Wang and Wang, 2016).

The efflux of xenobiotics and their conjugates from the cells occurs through diverse membrane transporters such as the ATP-binding cassette (ABC) proteins. This function confers the multidrug resistance (MDR), formerly described for chemotherapy-resistant cancer cells (Roninson et al., 1984; Gros et al., 1986), and the multixenobiotic resistance (MXR), defined by Kurelec (1992) for aquatic animals. As well as in mammals, the physiological roles of fish ABC proteins have been extensively studied in liver and kidney tissues (see reviews by Bard, 2000; Sturm and Segner, 2005; Ferreira et al., 2014; Luckenbach et al., 2014). In contrast, the studies on fish intestinal ABC proteins are still scarce and have not been reviewed so far.

This work summarizes physiological, molecular, immunochemical, and toxicological information on fish intestinal ABC proteins. We aim to update and integrate the current knowledge on fish intestinal ABC proteins concerning the organ’s physiology and responses to environmental threats.

## The Gastrointestinal System of Fish

Fish are classified into three groups: Agnatha (jawless fishes: hagfish and lampreys), Chondrichthyes (cartilaginous fishes), and Osteichthyes (bony fishes), among which Teleostei is the most abundant and diverse group (Betancur et al., 2017; Mikalsen et al., 2021). Most studies on ABC proteins focus on teleosts and chondrichthyans and lampreys to a lesser extent.

**FIGURE 1:**
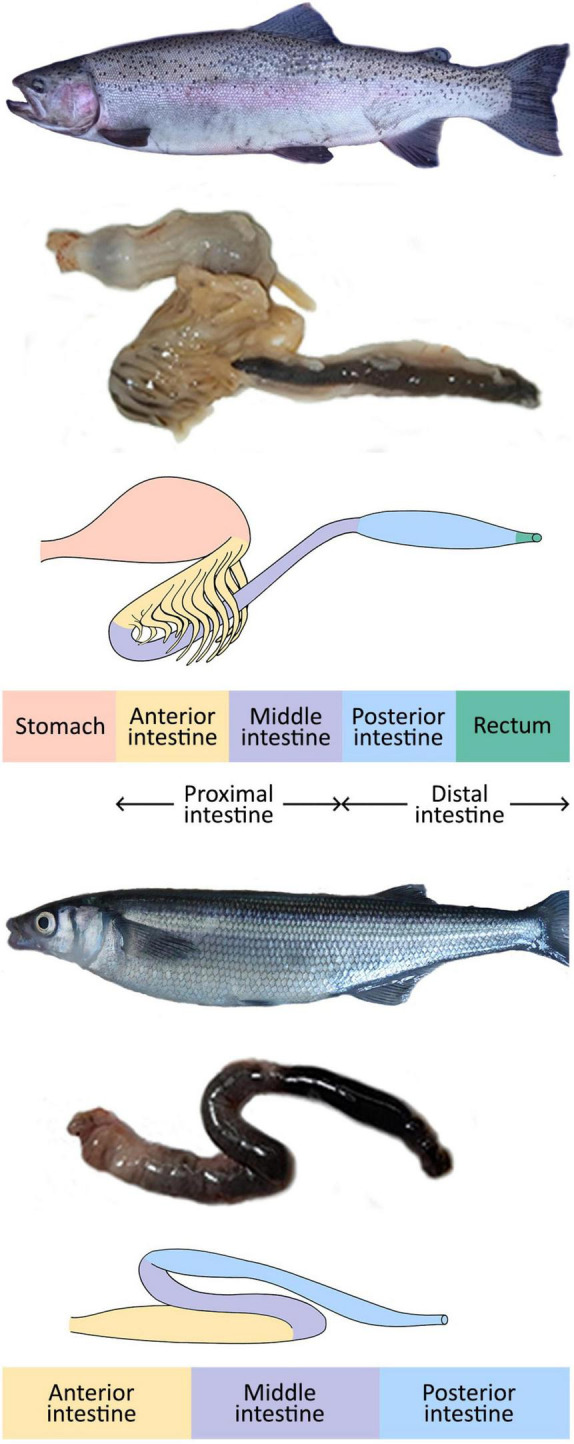
Rainbow trout (*Oncorhynchus mykiss*) and Patagonian silverside (*Odontesthes hatcheri*) (top and bottom, respectively) with a detail of their gastrointestinal tracts.

The fish GI consists of four regions: the headgut (the mouth and pharynx), foregut (the esophagus and stomach, where chemical digestion begins), midgut (the intestine, which completes chemical digestion and most of the absorption), and hindgut (the posterior region, including the rectum) (Wilson and Castro, 2011). The anatomy of fish intestines varies among species, and different authors describe it with different terminologies. For example, some authors use the term “gut” to refer to the GI, while others use it as a synonym of the intestine, making it difficult to compare studies. This review will refer to the intestine and its parts only with “intestine” (Figure 1).

The limit between intestinal portions is not always externally visible. However, histological features related to specific intestinal functions allow their identification (Grosell et al., 2011; Brugman, 2016). Lampreys, chondrosteans, chondrichtheans, and dipnoids have short intestines with the mucosa and submucosa folded to form a spiral valve, which increases the absorption surface area and delays the food passage. In addition, mucosal folds increase the surface area of the spiral folds (Wetherbee and Cortés, 2004; Wilson and Castro, 2011). At the distal end of the GI, chondrichtheans possess a salt-secreting structure, the rectal gland.

The stomach is present in most jawed vertebrates, including bony fishes. However, some teleosts have lost this structure and the genes encoding H^+^/K^+^-ATPase and pepsinogens (Koelz, 1992; Castro et al., 2013). In agastric fish, such as the zebrafish (*Danio rerio*), the intestine consists of an anterior portion or intestinal bulb, middle, and posterior intestine. The intestinal bulb has an enlarged lumen but lacks gastric glands and pylorus, and its pH is not lower than 7.5 (Brugman, 2016 for a review). In the anterior and middle portions, the presence of folds, digestive enzymes, and the expression of genes involved in lipid metabolism and solute transport indicate digestive and nutrient absorption functions. The posterior intestine mucosa has short folds and mediates water absorption (Brugman, 2016 for a review). Figure 1 shows a scheme of the GI of an omnivorous agastric fish, the Patagonian silverside, *Odontesthes hatcheri* (Bieczynski et al., 2016).

Most carnivorous fish, e.g., salmonids, possess a stomach with acid digestion enzymes. Their intestine consists of two portions, the anterior, which often bears numerous pyloric ceca that increase the surface area for digestion and absorption, and the posterior portion (e.g., Rust, 2003; Verdile et al., 2020). However, other authors (e.g., Kamalam et al., 2020) recognize three segments: the anterior with the pyloric ceca; the middle and posterior portions, which end in the rectum (Figure 1).

## ABC Proteins

The general characteristics, structure, function, substrates, and regulation of the proteins of the ABC superfamily are extensively described in the literature (e.g., Holland et al., 2003; Chan et al., 2004; Stolarczyk et al., 2011; Luckenbach et al., 2014; Thomas et al., 2020 for reviews). All these proteins have a highly conserved nucleotide-binding domain, NBD (or ATP binding cassette, ABC), which catalyzes the hydrolysis of ATP, coupling the energy released in this reaction to other cellular functions such as molecule transport. Their basic structure consists of a hydrophobic integral membrane domain (IM), also called transmembrane domain (TMD), and a hydrophilic cytoplasmic region, the NBD. The full transporters from this superfamily are composed of four structural domains, two IMs and two NBDs, while half transporters possess one of each domain.

According to their function, ABC proteins can be importers, exporters, or proteins with other roles such as DNA repair. Most eukaryotic ABC proteins are active exporters. In this context, exportation means transporting a molecule from the cytosol into another compartment, either extracellular or intraorganellar (Dassa, 2003). There are seven mammalian ABC subfamilies (ABCA-G) (Dean et al., 2001), two subfamilies identified in non-mammalian organisms (ABCH in insects and fish; ABCI in plants), and ABCJ, a new subfamily suggested for the mosquito *Aedes aegypti* (Verrier et al., 2008; Figueira-Mansur et al., 2020).

This review follows the Zebrafish Nomenclature Guidelines for the ABC gene and protein designation (ZFIN Zebrafish Nomenclature Conventions, 2019). For example, for zebrafish gene/protein: abcc/Abcc, and ABCC/ABCC for humans. Finally, we use capital letters to refer to ABC proteins in general or ABC subfamilies.

### Physiological Role of ABC Proteins

The phylogenetic relation of ABC genes shows the conservation of critical functions among species and some species- or lineage-specific functions (Annilo et al., 2006). There is a vast literature about the ABC functionality in humans and mammalian models because many human diseases are related to ABC failures (Dean et al., 2001). In fish and other organisms, there is still little knowledge on this subject. This section introduces the known physiological functions of each ABC protein in mammals and compares them with the available information on fish ABC proteins.

#### ABCA

This subfamily is related to cholesterol and lipid transport (Klaassen and Aleksunes, 2010; Hedditch et al., 2014; Szakacs and Abele, 2020). ABCA1 participates in the efflux to the plasma and maturation of cholesterol high-density lipoproteins (HDL) and transports other lipids and oxysterols (Venkateswaran et al., 2000; Brunham et al., 2006; Tam et al., 2006). ABCA2 is present in lysosome-related organelles, and its expression correlates with genes involved in cholesterol homeostasis (Szakacs and Abele, 2020). ABCA3 takes part in the regulation of pulmonary surfactants (Szakacs and Abele, 2020; Wambach et al., 2020). ABCA4 transports retinoid-lipid complexes (Cremers et al., 2020), and ABCA5 and ABCA7 regulate the amyloid-beta peptide production and transport (Fu et al., 2015; Aikawa et al., 2018).

Fish Abca1 is critical for assembling the HDL by transporting cholesterol from the cell to lipid-poor apoA-I. Its defect is responsible for aberrant angiogenesis in zebrafish (Fang et al., 2013, 2014 for a review). Kortner et al. (2014) have detected Abca1 in the pyloric caeca of the Atlantic salmon (*Salmo salar*), and Liu et al. (2016) report the RNA expression of abca1 and abca2 in the common carp intestine.

#### ABCB

The most studied ABC protein, ABCB1 (MDR1, Pgp), transports lipophilic compounds such as steroid hormones, and is related to toxic metabolites and xenobiotic efflux from the cytosol and is essential in the MDR phenotype (Gottesman et al., 1996; Chan et al., 2004). ABCB1, together with ABCC2 and ABCG2, has apical localization in polarized cells, forming a defense barrier against toxic compounds and therapeutic drugs. ABCB2 and ABCB3 (also named TAP1 and TAP2) are associated with antigen processing (Hinz and Tampé, 2012); ABCB4 (MDR2/3) is also apically located and transports phosphatidylcholine into the bile and also some drugs with similar substrate affinity as that of ABCB1 (Chan et al., 2004; Borst, 2020). ABCB5 is a marker of dermal cells with immunoregulatory functions (Schatton et al., 2015) and could be involved in melanogenesis (Chen et al., 2005). ABCB6 and ABCB7 are mitochondrial proteins; the former is a porphyrin transporter and takes part in the cellular defense against cadmium and arsenic (Boswell-Casteel et al., 2017; Wachnowsky et al., 2018; Rakvács et al., 2019). ABCB10 is a mitochondrial transporter related to hemoglobinization (Seguin et al., 2017). ABCB11, also called bile salt export pump (BSEP) or sister *P*-glycoprotein (Spgp), is apically located in hepatocytes and enterocytes and is critical for bile secretion. Its mutation is associated with cholestasis (Nayagam et al., 2020).

Abcb1 (Pgp) is also one of the most studied ABC proteins in fish and aquatic invertebrates. Fischer et al. (2013) have shown that, in zebrafish, two genes coding for apical membrane proteins with Pgp characteristics formerly recognized by Annilo et al. (2006) as abcb1a and abcb1b correspond to abcb5 and abcb4, and that abcb4 is the functional homolog of human ABCB1 in this species. Later studies show similar results for other fish species (see section “ABCB” for details). Like mammalian ABCB1 proteins, fish Abcb4 plays an essential role in extruding toxic compounds as a part of the MXR system (Kurelec, 1992; Bard, 2000; Sturm and Segner, 2005; Luckenbach et al., 2014 for reviews). Fish Abcb5 has a different function than the mammalian protein. It is another apical solute transporter related to xenobiotic efflux (Kropf et al., 2020; Robey et al., 2021). The zebrafish bile acid cyprinol sulfate induces the transcription of abcb5 in zebrafish liver (Reschly et al., 2007), suggesting potential roles of Abcb5 related to biliary excretion. Zebrafish Abcb3 and Abcb7 are mitochondrial transporters involved in heavy metal detoxification (Lerebours et al., 2016). Abcb7 participates in iron and fatty acid metabolism (Miyake et al., 2008). Abcb10, a mitochondrial ABC transporter associated with the response to oxidative stress, was detected in zebrafish muscle brain, gill, and intestine (Sabri et al., 2012). Like its mammalian counterparts, fish Abcb11 has an essential role in bile secretion (Ellis et al., 2018).

#### ABCC

This is a large subfamily that includes full transporters with diverse functions like ion transport, cell surface receptors, and detoxification of toxic compounds. In mammals, ABCC1 to 5 (MRP1-5) pump a structurally diverse array of endogenous substrates (leukotrienes, estrogen conjugates, bile salts, bilirubin, estradiol-17, organic anions, folic acid, cyclic nucleotides, among others) and xenobiotics out of the cells (Chan et al., 2004; He et al., 2011; Slot et al., 2011 for review). Among these proteins, only ABCC2 is apical, while the others are basolateral in all tissues, except ABCC4, which is apical in the kidney (Van Aubel et al., 2002; De Waart et al., 2012). ABCC6 exports nucleoside triphosphates, mainly ATP, from the hepatocytes to the bloodstream, and this activity is essential for a normal mineralization process (Szeri et al., 2021). It is also involved in the efflux of natural toxins and drugs (Chan et al., 2004). ABCC7 (cystic fibrosis transmembrane conductance regulator, CFTR) is an ATP-sensitive chloride channel mutated in patients with cystic fibrosis (Meng et al., 2018). ABCC8 and ABCC9 (SUR1 and SUR2) are sulfonylurea receptors; ABCC8 forms part of the structure of the ATP-sensitive potassium channel (K-ATP). ABCC10, ABCC11, and ABCC12 have been less studied, although they seem to be involved in drugs and endogenous compounds transport (Chan et al., 2004; Kruh et al., 2007).

In fish, the studies about the ABCC protective function against toxic substances concern mostly Abcc1 and Abcc2 but also Abcc3 to 5 (Klobučar et al., 2010; Cárcamo et al., 2011; Long et al., 2011a,b; Bieczynski et al., 2014, 2016; Painefilú et al., 2019, 2020; Lu et al., 2021; among others). Abcc6 is involved in the regulation of tissue calcification, e.g., scales mineralization (Parreira et al., 2018; Sun et al., 2021). Interestingly, this gene is present in bony vertebrates but not in elasmobranches, agnathans, or invertebrates (Parreira et al., 2018). The chloride channel Abcc7 has ionic and osmotic regulation functions in elasmobranches and bony fishes (Evans, 2010; Forrest, 2016 for reviews). Zebrafish abcc8 and abcc9 correspond to mammalian SUR1 and SUR2. Abcc8 is also part of ATP-sensitive potassium channels (KATP) (Zhang et al., 2006). The RNA expression of abcc9 is associated with cardioprotective sarcolemmal KATP currents in hypoxic fish (Cameron et al., 2013).

#### ABCD

These transporters are present in the peroxisomal (ABCD1, ABCD2, and ABCD3) and the endoplasmic reticulum and lysosomal (ABCD4) membranes (Tawbeh et al., 2021 for a review). Mammalian ABCD1 and ABCD2 export long- and very-long-chain fatty acids (VLCFA) or their CoA-derivatives into peroxisomes. ABCD3 participates in transporting branched-chain acyl-CoA into peroxisomes, while ABCD4 is related to vitamin B12 translocation from lysosomes to the cytosol (Baker et al., 2015). In humans, genetic defects of ABCD1 and ABCD3 are associated with two genetic disorders, the X-linked adrenoleukodystrophy (ALD) and the congenital bile acid synthesis defect 5, respectively (Tawbeh et al., 2021 for a review).

In zebrafish, abcd1 mutant individuals show elevated VLCFA levels, similar to human patients with ALD (Strachan et al., 2017). In turn, abcd4 knockdown zebrafish develop vitamin B12-deficiency anemia (Choi et al., 2019).

#### ABCE-F

These proteins are related to ribosome metabolism and are essential in the translation process. In most genomes, this subfamily only includes ABCE1, although two paralogs (abce1 and abce2) exist in some plant, fish, and mosquito species (Navarro-Quiles et al., 2018 for a review). A non-functional abce1 is associated with lethal and slow growth phenotypes in zebrafish embryos (Amsterdam et al., 2004; Navarro-Quiles et al., 2018 for a review). Similarly, ABCF members are associated with ribosome assembly or protein translation (Annilo et al., 2006). However, as far as we know, there are no functional studies on these proteins in fish.

#### ABCG

These proteins are involved in the transport of lipids and sterols, maintaining lipid homeostasis in cells, as well as in xenobiotic defense (Kerr et al., 2021 for a review). There are five ABCG-transporters (ABCG1, ABCG2, ABCG4, ABCG5, and ABCG8) in humans. ABCG1 exports cholesterol through the basolateral membrane to phospholipid-rich nascent HDL particles, oxysterols, and cholesterol synthesis intermediates in various cell types. In addition, ABCG1 plays a role in regulating inflammation, and it participates in lipid storage and exportation out of adipocytes. The breast cancer resistance protein (ABCG2-BCRP) is expressed in “barrier” sites, e.g., the apical membrane of enterocytes, and is related to limiting the uptake of drugs and xenobiotics into the bloodstream. Also, it is involved in the non-renal clearance of uric acid and the transport of endogenous substrates such as conjugated steroid hormones. ABCG4 is involved in the transport of cholesterol, oxysterols, and cholesterol-synthesis intermediates. ABCG5 and ABCG8 mediate the intestinal and biliary efflux of neutral sterols, such as cholesterol, plant sterols, and shellfish defense sterols. These proteins are half-transporters that become functional by forming a heterodimer that translocates to the apical membrane (Graf et al., 2003; Kerr et al., 2021 for a review).

Reports on fish Abcg1 are available only for zebrafish lipid metabolism and related human diseases (atherosclerosis and angiogenesis) (Fang et al., 2013, 2014 for a review). Abcg2 is the most studied member of this subfamily in fish as an essential protein concerning xenobiotics detoxification (Paetzold et al., 2009; Kropf et al., 2016; Zaja et al., 2016, among others). (Zhang L. et al., 2020) characterized zebrafish abcg5 and abcg8. The proteins codified by these genes are present in zebrafish embryos and adult individuals (mainly in the liver and intestine). These authors report that the products of zebrafish abcg5 and abcg8 transfected in a cell system localize to the cell membrane only when both genes are co-expressed. A study by Zhu et al. (2018) shows that rainbow trout (*Oncorhynchus mykiss*) fed with a plant-based diet (poor in cholesterol) suffer abcg8 (among other genes) down-regulation, probably as a mechanism to maintain cholesterol homeostasis.

#### ABCH

This subfamily was first identified in the *Drosophila melanogaster* genome and is present in the sea urchin and in all the arthropod genomes sequenced up to know, but not in fungi, plants, or mammals (Annilo et al., 2006; Sturm et al., 2009; Popovic et al., 2010; Dermauw and Van Leeuwen, 2014). Among fish, the abch gene appears only in the zebrafish and in the green spotted pufferfish (*Tetraodon nigroviridis*) (Luckenbach et al., 2014).

## ABC Proteins in Fish

### Whole-Genome Analyses

The superclass Agnatha is represented now by lampreys and hagfishes. Ren et al. (2015) identified 37 ABC genes: seven abca, ten abcb, ten abcc, three abcd, one abce, three abcf, and three abcg, in a genome-wide ABC gene survey in the sea lamprey (*Petromyzon marinus*) and the Japanese lamprey (*Lethenteron japonicum*). Besides, by high throughput RNA sequencing in tissues from different developmental stages, these authors recorded a strong expression of abce and abcf genes plus the abcb7 and abcc5 genes in all or most analyzed tissues. Particularly in the intestine, the most highly expressed transporters were abce1, abcf1 and abcf2, followed by abcb1-like, abcb6, abcb7, abcb10, abcc2, abcc5, abcd2, and abcd3. In the parasite stage, the expression of abca12 transcripts was higher in the distal than in the proximal intestine. Interestingly, abcb1 was not present in this organ, but abcb1-like was highly expressed. The abcc4 and abcg2a transcripts were highly expressed only in the small parasite distal intestine.

After the first comprehensive zebrafish genome by Dean and Annilo (2005); Annilo et al. (2006) report that several duplication events and gene deaths have occurred throughout the evolution of vertebrate ABC genes. These changes include ancient events such as the apparent whole-genome duplication in fish and more recent events such as individual ABC duplications. These authors identify fifty-two zebrafish ABC genes (nine abca, fourteen abcb, thirteen abcc, five abcd, one abce, three abcf, nine abcg, and one abch gene). They also report more frequent duplication events in abca, abcb full-transporters, and abcg than in genes encoding abce, abcf, abcb half transporters, and abcc. Liu et al. (2013) identify a set of fifty ABC genes in the channel catfish (*Ictalurus punctatus*) genome, which belong to seven subfamilies: nine abca, twelve abcb, twelve abcc, five abcd, two abce, four abcf, and six abcg genes, but no abch. Jeong et al. (2015), through bioinformatics-aided *in silico* analyses, have characterized 50 putative ABC transporters in the marine medaka (*Oryzias melastigma*), including one putative Abch gene. From a phylogenetic analysis, these authors have determined that these ABC genes are members of the eight ABC subfamilies, including ten abca and ten abcb, thirteen abcc, four abcd, one abce, three abcf, eight abcg, and one abch.

Posteriorly, Liu et al. (2016) identified 61 ABC genes representing seven subfamilies in the common carp (*Cyprinus carpio*) genome, including eleven abca, six abcb, nineteen abcc, eight abcd, two abce, four abcf, and eleven abcg. These authors also studied the expression of each ABC gene in the brain, gills, kidney, spleen, heart, and intestine by qPCR. Most of the ABC transcripts were ubiquitous, but their expression levels were tissue-specific. Interestingly, abca2 was detected only in the intestine. Transcripts of the remaining abca and abcb genes identified in the genome were also present in this organ, except abca12 and abcb5-1. All the abcc members were expressed in the intestine with variable intensity, except abcc7, abcc8, and abcc12-1 genes that were not detected. Except for the high expression of abcd4-2, the abcd genes were absent or weakly expressed in the intestine. The abce and f subfamilies were also present in the intestine, showing abce1-2 the highest expression levels. Finally, most abcg genes had weak expression in the intestine except for abcg5, which was abundant in all tissues.

### Expression and Physiological Role of Fish Intestinal ABC Proteins

**FIGURE 2:**
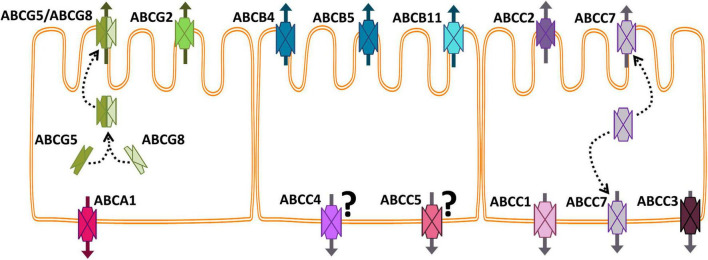
Schematic representation of the intestinal epithelium showing the most studied fish ABC proteins localized. Arrows indicate the direction of the substrates’ transport. The question mark next to ABCC4 and ABCC5 indicates that the basolateral location of these proteins in the intestine has not been confirmed yet for fish.

In this and the following sections, we will focus on the expression, function, toxicology, and regulation of the ABC proteins that have been studied so far in the fish intestine. Most of the earlier studies, particularly those based on anti-mammalian protein antibodies, use a functional terminology, e.g., Pgp, MDR, MRP. In cases in which the information provided or the technique applied by the original authors does not allow to identify specific abc/Abc gene/proteins, we will keep the terms Pgp or MRP of the original paper. Table 1 summarizes all the studies on fish intestine ABC proteins, and Figure 2 shows the localization of the most studied ABC proteins in the fish enterocytes.

**TABLE 1:** Studies on fish intestine ABC proteins.

Species	ABC protein	Methodology	References
*Anguilla japonica*	Abcc7	F	Ando and Takei, 2015
	Abcc7	IHC, M	Wong et al., 2016
*Cyprinus carpio*	ABCA - ABCH	M	Liu et al., 2016
*Danio rerio*	Abcb4	B, F, M, T, WISH	Lu et al., 2015
	Abcb4; Abcb5	F, IHC, M, MF, T, WB	Robey et al., 2021
	Abcc1 to 5	M, F	Moraes et al., 2020
	Abcc2	F, IHC, M, MF, T, WISH	Long et al., 2011c
	Abcc4	B, F, IHC, M, MF, T, WB, WISH	Lu et al., 2014
	Abcc4	B, F, M, T, WB, WISH	Lu et al., 2021
	Abcc5	B, M, MF, T, WISH	Long et al., 2011a
	Abcg2	M	Kobayashi et al., 2008
	Abcg2	M, T	Zhang Q.L. et al., 2020
	Abcg5, Abcg8	B, IHC, M, MF, T, WB	Zhang L. et al., 2020
	Abch1	M	Popovic et al., 2010
*Dicentrarchus labrax*	Abcc7	IHC, WB	Bodinier et al., 2009a
	Abcc7	DB, IHC, M	Bodinier et al., 2009b
	Abcc7*	F, M	Alves et al., 2019
*Fundulus heteroclitus*	Pgp	IHC, M, WB	Cooper, 1996
	Pgp	IHC	Bard et al., 2002
	Abcc7	F, M	Singer et al., 1998
	Abcc7	F, IHC, WB	Marshall et al., 2002
*Gobiocypris rarus*	Pgp, Abcb11, Abcc1, Abcc2 and Abcg2	M	Yuan et al., 2014
*Ictalurus punctatus*	Pgp	IHC, T	Kleinow et al., 2000
	Pgp	F, WB	Doi et al., 2001
*Lethenteron japonicum*	ABCA-ABCG	M	Ren et al., 2015
*Odontesthes hatcheri*	Abcc2, basolateral ABCCs	B, F, T	Bieczynski et al., 2016
*Oncorhynchus mykiss*	Abcb4, Abcb11, Abcc1-5, Abcg2	M	Lončar et al., 2010
	Abcb4, Abcc1	M, T	Cárcamo et al., 2011
	Abcb4, Abcb5	M, T	Love et al., 2021
	Abcc2, basolateral ABCCs	B, F, T	Bieczynski et al., 2014
	Abcc2, basolateral ABCCs	B, F, M, T	Painefilú et al., 2019
	Abcc2, basolateral ABCCs	B, F, T	Painefilú et al., 2020
	Abcb4, Abcc2	M, T	De Anna et al., 2021
*Opsanus beta*	Abcc7	F, IHC, M	Ruhr et al., 2014
	Abcc7	F, M	Ruhr et al., 2015
	Abcc7	F, M	Ruhr et al., 2016
	Abcc7*	F, IHC, WB	Ruhr et al., 2018
*Oreochromis mossambicus*	Abcc7	IHC, M	Li et al., 2014
*Oreochromis niloticus*	Abcb11, Abcc1, Abcc2, Abcg2	M, T	Costa et al., 2012
	Pgp	IHC, WB	Costa et al., 2013
*Petromyzon marinus*	Abcb9	M	Uinuk-Ool et al., 2003
	ABCA – ABCG	M	Ren et al., 2015
	Abcc7	F, IHC, M, WB	Cui et al., 2019
*Poecilia reticulata*	Pgp	IHC	Hemmer et al., 1995
*Raja erinacea*	Abcc2	M, IHC, WB	Cai et al., 2003
*Rasbora sarawakensis*	ABCA – ABCH	M	Lim et al., 2021
*Salmo salar*	Abca1, Abcg5	M	Kortner et al., 2014
*Scophthalmus maximus*	Pgp	M, WB	Tutundjian et al., 2002
*Sparus aurata*	Abcc7*	F, IHC, M	Gregório et al., 2013
*Squalus acanthias*	Abcc2	F, IHC	Miller et al., 1998
	Abcc7**	IHC, M, WB	Marshall et al., 1991
	Abcc7**	F, IHC, WB	Lehrich et al., 1998
	Abcc7**	F, M	Bewley et al., 2006
	Abcc7**	F, M, T	Ratner et al., 2006
	Abcc7**	F, M	Stahl et al., 2012
	Abcc7**	F, M	De Jonge et al., 2014
	Abcc7**	F	Telles et al., 2016
	Abcc7**	F	Neuman et al., 2018

*B, biochemistry; DB, dot blot; F, functional assays; IHC, immunohistochemistry or immunofluorescence; M, molecular biology; MF, molecular biology – functional; T, toxicology; WB, western blot; WISH, whole-mount in situ hybridization.*

Asterisks indicate studies on the intestine and rectum (*) or rectal gland (**).

#### ABCA

In mammalian enterocytes, ABCA1 transports sterols and lipids through the basolateral membrane contributing to the formation of plasmatic HDL (e.g., Brunham et al., 2006). In fish, Liu et al. (2016) have reported the presence of abca1 and abca2 in the common carp intestine. Furthermore, in the Atlantic salmon (*Salmo salar*), adding cholesterol to a plant-based diet increases the abca1 and abcg5 RNA expression in the pyloric caeca, the primary site of lipid absorption (Kortner et al., 2014). Since abca1 is basolateral and abcg5 is an apical transporter, this induction suggests that *S. salar* pyloric abca1 and abcg5 (as Abcg5/8 heterodimer, see section “ABCG and ABCH”) regulate the intestinal cholesterol flux by favoring absorption or excretion, respectively, according to the cholesterol level in the diet. A possible protective role of this regulatory mechanism by exporting cytotoxic oxysterols deserves investigation.

#### ABCB

Phylogenetic analysis shows that fish abcb1, abcb4, and abcb5 are closely related genes, indicating a shared common ancestor during chordate evolution (Annilo et al., 2006; Fischer et al., 2013; Luckenbach et al., 2014). Formerly, Annilo et al. (2006) recognized two abcb1 genes, abcb1a and abcb1b, and no abcb4 in zebrafish. They stated that ABCB4 appeared in the mammalian lineage; thus, it was absent in fish. Later, Fischer et al. (2013) showed that, in zebrafish, abcb1a and abcb1b corresponded to abcb5 and abcb4, respectively. The whole-genome analyses presented in the previous section of this review show that at least one of these transporters (abcb1, abcb4) is present in any genome. For example, the zebrafish and the rainbow trout have abcb4 and abcb5 but not abcb1 (Fischer et al., 2013; Luckenbach et al., 2014; Kropf et al., 2020).

Zebrafish abcb4 is highly expressed in the intestine and is the most similar fish abcb to human ABCB1 (Fischer et al., 2013; Robey et al., 2021). However, it is unclear whether the genes described in pioneering works as abcb1, abcb1-like, Mdr1, and Pgp correspond to abcb1 or abcb4. Consequently, we will use Pgp to denote genes not clearly identified as abcb1 or abcb4.

Chan et al. (1992) described for the first time the presence and the molecular structure of two Pgp genes in the winter flounder *Pleuronectes americanus*. From then on, studies on Abcb transporters in fish tissues have increased thanks to the availability of different methodologies, such as immunohistochemistry, immunoblot, molecular biology, model substrate transport, and ATPase activity assays.

It is important to notice that the antibodies used in the studies described herein have been raised against mammalian proteins. Therefore, their specificity toward fish representatives of the different ABC proteins may not be complete, or the binding to the target protein could fail. Considering this constraint, Hemmer et al. (1995) detected ABCB proteins in the guppy (*Poecilia reticulata*) by immunohistochemistry with four different anti-mammalian ABCB1 antibodies: C219, which recognizes ABCB1, ABCB4, and ABCB11; C494, which recognizes ABCB1, but not ABCB4, JSB-1 (which binds to a conserved epitope of ABCBs), and mdr(Ab-1), similar to C219. The intestinal epithelium showed similar labeling intensity with all the antibodies, while the subjacent smooth muscle showed negative results. Another early work by Cooper (1996) reports high protein expression of Pgp in the liver and lower-level in the intestine of the killifish (*Fundulus heteroclitus*) by immunoblot with the antibody C219.

Similarly, Kleinow et al. (2000) identified a Pgp protein in the apical border of the intestinal epithelium of the channel catfish intestine by immunohistochemistry with C219. The previous oral exposure of these fish to the human Pgp agonist, vincristine, or β-naphthoflavone (an agonist of the aryl hydrocarbon receptor, AhR) increased the Pgp immunostaining. Tutundjian et al. (2002) studied the expression pattern of a DNA sequence that was 73% identical to several mammalian ABCB sequences in the turbot (*Scophthalmus maximus*). They detected the highest mRNA expression in the brain, intestine, and kidney. In contrast, their Western blot studies with C219 showed higher protein expression in the kidney and brain than in the intestine, heart, and gills.

Costa et al. (2013) have reported that C494 (specific for ABCB1) labels enterocytes and vascular endothelium of the Nile tilapia (*Oreochromis niloticus*). They have also found that C219 labels the apical membrane of the enterocytes. Umans and Taylor (2012) studied Pgp functions in the zebrafish larvae blood-brain barrier and intestine. Concerning the intestinal mucosa, these authors showed positive immunostaining with C219. They confirmed the detected protein’s functionality by exposing the larvae to the mammalian ABCB1 fluorescent substrate doxorubicin in water and measuring its accumulation in the intestinal lumen. Table 2 details the antibodies used in the papers cited in this review.

**TABLE 2:** Antibodies utilized to detect fish intestine ABC proteins.

Antibody	Protein detected	Technique	Species	References
C219 (m)	Human MDR1 (ABCB1), MDR2/3 (ABCB4), and BSEP (ABCB11)	IHC	*Poecilia reticulata*	Hemmer et al., 1995
		IHC, WB	*Fundulus heteroclitus*	Cooper, 1996
		IHC	*Ictalurus punctatus*	Kleinow et al., 2000
		WB	*Ictalurus punctatus*	Doi et al., 2001
		WB	*Scophthalmus maximus*	Tutundjian et al., 2002
		IHC, WB	*Fundulus heteroclitus*	Bard et al., 2002
		IF	*Danio rerio*	Umans and Taylor, 2012
		IF, WB	*Oreochromis niloticus*	Costa et al., 2013
		IF	*Danio rerio*	Robey et al., 2021
mdr(Ab-1) (p)		IHC	*Poecilia reticulata*	Hemmer et al., 1995
C494 (m)	Human MDR1 (ABCB1)	IHC	*Poecilia reticulata*	Hemmer et al., 1995
		IF, WB	*Oreochromis niloticus*	Costa et al., 2013
JSB-1 (m)	Human MDR	IHC	*Poecilia reticulata*	Hemmer et al., 1995
EAG15 (p)	Rat ABCC2	IHC	*Squalus acanthias*	Miller et al., 1998
Anti-skate Abcc2 (p)	Skate Abcc2	IF	*Raja erinacea*	Cai et al., 2003
Anti-CFTR (m)	Human CFTR (ABCC7)	IHC, WB	*Squalus acanthias*	Marshall et al., 1991
		IF, WB	*Fundulus heteroclitus*	Marshall et al., 2002
		IF, WB	*Dicentrarchus labrax*	Bodinier et al., 2009a
		DB, IF	*Dicentrarchus labrax*	Bodinier et al., 2009b
		IF	*Sparus aurata*	Gregório et al., 2013
		IF	*Oreochromis mossambicus*	Li et al., 2014
		IF	*Opsanus beta*	Ruhr et al., 2014
		IF	*Opsanus beta*	Ruhr et al., 2018
Anti-lamprey CFTR (p)	Lamprey Cftr (Abcc7)	IHC, WB	*Petromyzon marinus*	Cui et al., 2019
MAB25031 (m)	Human CFTR (ABCC7)	IHC	*Anguilla japonica*	Wong et al., 2016
R3195 (p)	Rodent CFTR (ABCC7)	IF, WB	*Squalus acanthias*	Lehrich et al., 1998

*DB, dot blot; IF, immunofluorescence; IHC, immunohistochemistry; m, monoclonal; p, polyclonal; WB, western blot.*

Fischer et al. (2013) report that unlike the mammalian ABCB4 (which has a different substrate affinity), zebrafish Abcb4 is functionally similar to the mammalian ABCB1. Besides, these authors have examined whether Abcb4 or Abcb5 participate in xenobiotic efflux through rhodamine B and calcein accumulation assays performed in morpholino-knockdown embryos. They have concluded that the changes in rhodamine B or calcein accumulation occur specifically in Abcb4 knockdown; thus, Abcb4, but not Abcb5, acts as an efflux pump for those substrates. Using recombinant Abcb4 zebrafish protein, these authors have confirmed the detoxification function of Abcb4 by studying the effects of fluorescent dyes and well-known human ABCB1 substrates on ATPase activity. Bieczynski et al. (2021) established an automated microscopy-based rhodamine B dye accumulation assay for studying the interaction between several well-known human ABC substrates/inhibitors and aquatic pollutants with Abcb4 in zebrafish embryos; and also analyzed the effects of these substances on the ATPase activity of recombinant zebrafish Abcb4. These two assays confirmed that Abcb4 functions as a broad-spectrum multixenobiotic efflux transporter in zebrafish, as previously described by Fischer et al. (2013). However, the results from both tests were not always concordant. In adult zebrafish, Lu et al. (2015) found the highest abcb4 mRNA expression in the intestine, followed by the liver, and then muscle > gill > eye > ovary > testis > heart > brain > kidney, suggesting a vital role of Abcb4 transporters in the barrier function of the intestinal mucosa.

Gordon et al. (2019) have shown that zebrafish Abcb5 has Pgp functions but different substrate affinity from Abcb4. They detected high RNA expression of abcb5 in proton pump-rich ion-transporting cells (HR ionocytes) of the epidermis of zebrafish embryos through single-cell transcriptomics. HR cells accumulate several fluorescent ABC substrates but not rhodamine B, and the addition of ABCB1 and ABCC inhibitors significantly increase this accumulation. These results suggest a protective role of the epidermis ionocytes in part through Abcb5-mediated efflux of toxic molecules in early stages with not completely developed detoxification organs.

Robey et al. (2021) provide further information on the function and expression patterns of abcb4 and abcb5 in zebrafish. Immunofluorescence and *in situ* hybridization show that abcb4/Abcb4 is more expressed than abcb5/Abcb5 in the blood-brain barrier, intestine, and liver. Additionally, using transfected cells, they have compared both Abcbs’ substrate specificity through a cytotoxicity assay with known human ABCB1 substrates. They report that both transporters confer similar resistance levels as human ABCB1 to vinblastine and paclitaxel, although Abcb4 confers higher resistance to bisantrene, mitoxantrone, and doxorubicin than Abcb5. Abcb4 and Abcb5 share a similar affinity for the fluorescent ABCB1 substrates calcein-AM, rhodamine 123, BODIPY-prazosin, Flutax, BODIPY-vinblastine, and TMRE. However, only Abcb4 transports the substrates LDS 751 and BODIPY-EDA. The set of ABCB1 specific inhibitors tested by these authors inhibits the efflux of most of these substrates by both, Abcb4 and Abcb5 to a similar extent. Finally, a high throughput screening with 90 cytotoxic human ABCB1 substrates led the authors to conclude that zebrafish Abcb4 is functionally homolog to human ABCB1.

In the same report, ATPase activity and molecular structure studies complete the analysis on the homologies of zebrafish Abcb4 and Abcb5. Both proteins partially differ from human ABCB1 in the drug-binding pocket; however, Abcb5 has more significant differences in amino acids relative to ABCB1 than Abcb4. This result could explain the difference in substrate specificity between these proteins.

Most studies on Abcb11 refer to the liver, where this protein localizes to the hepatocytes apical membrane and transports bile salts in the rate-limiting step of the hepatocyte bile secretion (Boyer and Soroka, 2021). The zebrafish genome predicts the presence of two co-orthologs of the human ABCB11, abcb11a, and abcb11b (Ellis et al., 2018). By whole-mount *in situ* hybridization, these authors have identified abcb11a as the predominant isoform in the intestine, while abcb11b RNA is specifically expressed in the liver.

#### ABCC

Lončar et al. (2010) analyzed the mRNA expression of a set of ABC transporters in rainbow trout tissues. They detected the presence of abcc1 to 5 in the anterior and posterior intestine, showing a much larger number of transcript copies of abcc3 than the others ABCC. However, the relative abundance of the different abcc transcripts is subject to modulation by endogenous or exogenous factors (see citation to Cárcamo et al., 2011 in section “Pesticides and Pharmaceuticals”).

Miller et al. (1998) showed that, besides its primary role in osmotic-ionic regulation, the rectal gland of the elasmobranches could actively excrete xenobiotics through an analog of ABCC2. These authors studied the transport of the fluorescent substrates sulforhodamine 101 and fluorescein methotrexate combined with known ABC substrates/inhibitors in isolated dogfish shark (*Squalus acanthias*) rectal gland tubule fragments. This transport was sensitive to cyclosporine A, chloro-dinitrobenzene (CDNB), and leukotriene C4 but not the Pgp inhibitor verapamil. Besides, they have confirmed the presence of Abcc2 in the luminal membrane of the rectal gland epithelial cells by immunohistochemistry with a polyclonal anti-mammalian ABCC2 antibody (Table 2). Posteriorly, Miller et al. (2002) described a hormonal regulatory pathway for the rectal gland Abcc2-mediated transport similar to that described by Masereeuw et al. (2000) for the killifish renal tubules. Endothelin-1 (ET1) inhibited sulforhodamine 101 transport through an ET_*B*_ receptor that induces protein kinase (PKC).

In the same paper, Miller et al. (2002) reported that the activation of the cAMP pathway by forskolin inhibits the luminal Abcc2-mediated transport by a PKA-independent mechanism. Applying a cAMP analog that does not activate PKA produced a similar effect, suggesting that cAMP could interact directly with the transporter, possibly as a competitive substrate. In this case, the transport of cyclic nucleotides by Abcc2 or other apical Abcc could be part of the modulation of apical ion transport proteins, thus regulating the rectal gland’s primary function.

Cai et al. (2003) studied the molecular structure and properties of the little skate (*Raja erinacea*) Abcc2 and found phylogenetic and structural similarities with the mammalian ABCC2. Their immunofluorescence studies with a specific anti-skate Abcc2 antibody localize this protein at the apical membrane of hepatocytes, kidney tubules cells, and small intestine enterocytes.

As for teleosts, a whole-mount *in situ* hybridization and qRT-PCR study in developing and adult zebrafish (Long et al., 2011c) shows that after 72 hpf, abcc2 has the highest expression in the intestine, followed by the kidney and the liver. In agreement, Fischer et al. (2013) report high RNA expression of abcc2 in rainbow trout intestinal cells (RTgutGC). In contrast, Lončar et al. (2010) detected abcc2 transcripts in the rainbow trout intestine but at lower levels than other ABC transporters such as abcg2 and abcb4.

Bieczynski et al. (2014) report the apical transport of the ABCC substrate DNP-SG in polarized preparations of rainbow trout middle and distal intestine. This transport is sensitive to the specific ABCC inhibitor MK571 suggesting the involvement of Abcc2. Painefilú et al. (2019) show that the previous exposure of rainbow trout to arsenic (as AsIII) for 48 h increases the rate of apical DNP-SG extrusion in intestinal preparations and that this effect coincides with the induction of abcc2 mRNA expression (more details in section “Toxicological Studies”).

Long et al. (2011a) suggest that zebrafish Abcc5 may be involved in tissue defense against xenobiotics and embryonic development and maintenance of normal physiological functions through the transport of cyclic nucleotides such as cGMP. Transcripts of this gene are ubiquitous in embryos, and since 96 hpf, abcc5 expression is evident in the intestine, among other organs. Besides, blockade of Abcc5 activity in embryos overexpressing an inactive Abcc5 form retards development and increases cGMP levels. In contrast, embryos that overexpress the typical Abcc5 show normal development and maintain a cGMP concentration similar to that of control embryos.

Sun et al. (2021) used abcc6a mutant zebrafish as a model to increase knowledge of the mechanisms underlying the human disease Pseudoxanthoma elasticum multisystem disorder (PXE). Besides the effects on other organs, these authors report the presence of abcc6 transcripts in the intestine muscularis external and lamina propria and weak expression in some enterocytes. They have found that abcc6aD1/D1 mutants have PXE-like symptoms, such as fibrosis and thickening of the muscularis external and lamina propria compared to wild-type fish, but not detectable calcification in the intestine.

Abcc7 (CFTR) is a chloride channel extensively studied in seawater teleosts, lampreys, and elasmobranchs concerning ion transport and water balance (Marshall et al., 2002, 2009; Grosell, 2006, 2011; Takei and Yuge, 2007; Bodinier et al., 2009a,b; Evans, 2010; Gregório et al., 2013; Ruhr et al., 2018; Cui et al., 2019). The intestine of seawater fish can switch from net ion absorption to net ion secretion by regulating the expression, activity, and localization of different channels and transporters, such as NKCC2, HCO3-/ Cl-, and Abcc7 in the enterocytes. These events are under endocrine control and respond to intracellular stimuli, which activate cyclic AMP and GMP pathways (Takei and Yuge, 2007; De Jonge et al., 2014; Ruhr et al., 2014, 2018; Ando and Takei, 2015; Forrest, 2016; Kinne et al., 2020). Pioneering works studied Abcc7 in the rectal gland of elasmobranchs. This model allowed the authors to know that the Abcc7 phosphorylation by the cAMP-PKA pathway stimulates its traffic to the apical membrane and the opening of the Abcc7 channel (Marshall et al., 2002; Evans, 2010; Forrest, 2016 for reviews). Nowadays, there are numerous studies on this transporter’s cellular location, function, and regulation by immunohistochemistry, molecular and electrophysiological techniques in teleosts, elasmobranchs and lampreys’ gills, opercular epithelium, skin, pancreas, intestine, and rectum (Marshall et al., 2002, 2005; De Jonge et al., 2014; Forrest, 2016; Ruhr et al., 2018; Cui et al., 2019). Table 1 summarizes the studies on abcc7/Abcc7 performed exclusively in the fish intestine, rectum, and rectal gland.

Wong et al. (2016) studied the structure and function of the abcc7 isoforms a and b in the Japanese eel by qPCR and immunohistochemistry. They reported that abcc7a (and its protein product Abcc7a) is the dominant isoform in the intestine and kidney. Its expression is higher in freshwater than in seawater. On the contrary, abcc7b/Abcc7b is present predominantly in the gills, and its expression augments in seawater conditions. Cui et al. (2019) examined an ancient abcc7 ortholog found in the sea lamprey, the earliest known abcc7. These authors studied the abcc7/Abcc7 distribution in the sea lamprey intestine by qPCR and immunoblot. In adults and larvae, they found high mRNA and protein expression in the distal portion of the gastrointestinal system.

#### ABCG and ABCH

In contrast with mammals, which have only one ABCG2, fish possess multiple copies of this gene. For example, there are two paralog genes (abcg2a and abcg2d) in the rainbow trout genome, and four predicted abcg2 (a-d) in the zebrafish genome (Annilo et al., 2006). These abcg2 paralogs are differentially expressed among zebrafish tissues. While abcg2a and abcg2b have a strong expression in the intestine, abcg2c is highly expressed in the gills, intestine, spleen, and kidney but not in the liver. In contrast, these authors could not find abcg2d expression in any tissue (Kobayashi et al., 2008).

Zaja et al. (2016) studied the properties of the rainbow trout Abcg2a by expressing the transporter in Sf9 insect cells and analyzing the effects of several toxic compounds on ATPase activity. They have obtained similar results to those obtained for mammalian ABCG2. The model ABCG2 activator, sulfasalazine, caused maximum ATPase activation at 100 μM. Curcumin, benzo (a) pyrene (BaP), and testosterone produced the highest activation (maximum effects with 1, 10, and 100 μM, respectively).

Matsumoto et al. (2020) have cloned and studied the distribution pattern of abcg2 in the marine pufferfish. They have detected two abcg2 genes with the highest RNA expression in the intestine, liver, and kidneys. Their phylogenetic analysis assigns the pufferfish abcg2 to the abcg2b gene paralog group. In addition, these authors report a hypoxia response element and an estrogen response element located upstream of the abcg2 sequence, suggesting that the transcriptional regulation of the pufferfish abcg2 responds to sex hormones and is induced by the hypoxia-inducible factor, as it occurs for the human ABCG2.

Abcg5 and Abcg8 proteins are present in the Atlantic salmon pyloric caeca and the zebrafish intestine (Kortner et al., 2014; Zhang L. et al., 2020). Besides a possible physiological role in biliary and intestinal transport of neutral sterols, reported for mammalian ABCG5/8, Zhang L. et al. (2020) suggest that zebrafish Abcg5/8 could transport organochlorine pesticides (see section “Toxicological Studies”).

Abch1 is a relatively recently discovered ABC transporter. Popovic et al. (2010) have detected abch1 through qPCR analysis in different zebrafish tissues, including the intestine. Jeong et al. (2015) report the presence of Abch1 in the marine medaka. These authors suggest that considering the moderate similarity in topology and tissue distribution pattern between Abch1 and ABCG transporters, Abch1 can be involved in sterol transport similar to Abcg1 or MXR defense, like Abcg2. However, there are no functional studies about fish intestine ABCG or ABCH transporters as far as we know.

## ABC Distribution Along the Fish Intestine

Lončar et al. (2010) studied the expression by qPCR of abcb4, abcb11, abcc1 to 5, and abcg2 in different rainbow trout tissues. In the proximal intestine, the expression of abcc3 was 2–3-fold higher than that of abcg2, followed by abcb11, abcb4, and abcc5 > abcc2 > abcc1 and abcc4. In contrast, the distal intestine showed the highest level of abcg2 and abcb4, followed by abcc3 and abcc2. The abcb4 expression was more than 7-fold higher in the distal than in the proximal part. Accordingly, immunohistochemical studies reported higher reaction with the anti-Pgp antibody C219 in the distal than in the proximal intestine of the channel catfish (Kleinow et al., 2000) and the killifish (Bard et al., 2002). In addition, Costa et al. (2013) reported increasing label intensity with another anti-Pgp antibody, C494, from the crypts to the tips of the intestinal folds in the Nile tilapia proximal intestine. These distribution patterns are similar to the reported for human and rodent intestinal ABCB1 mRNA and protein expression (Mottino et al., 2000; Chan et al., 2004; Müller et al., 2016).

Fischer et al. (2011) studied the expression of abcb4, abcb5, abcb11, abcc1 to 5, and abcg2 in seven rainbow trout permanent cell lines using qRT-PCR and functional assays (fluorescence substrate accumulation, combined with different ABC inhibitors). They found that transcripts of all the studied transporters were present in the seven rainbow trout cell lines, having abcc2 the highest expression. In the intestine cell line RTgutGC, the order of expression was: abcc2 > abcc3 = abcc1 > abcc5 > abcc4 = abcg2 > abcb4 > abcb 11 > abcb5. The ABC expression between the intestine and the RTgutGC model shows marked differences; especially, the cell line has higher abcc2 expression than the intestine. Besides, the most expressed transporters in the intestine, abcg2, and abcb4, show weak expression in the RTgutGC cell line (Lončar et al., 2010; Fischer et al., 2011). The differential transporter expression between tissues and cell cultures could lead to erroneous conclusions from functional or toxicological experiments. For example, Boaru et al. (2006) observed no effects of the cyanotoxin microcystin-LR (MCLR) on RTL-W1 and RTgutGCl cell lines (from rainbow trout’s liver and intestine, respectively), neither on HepG2 and CaCo2 (human), which was an unexpected result. Then, these authors analyzed the expression of OATP (organic anion-transporting polypeptides), the proteins by which MCLR would enter the cells in the RTL-W1 and RTgutGCl cell lines and rainbow trout primary hepatocytes. They detected no oatp mRNA expression in the rainbow trout cell lines. In contrast, freshly prepared primary hepatocytes expressed oatp mRNA and were sensitive to MCLR, but this expression decreased drastically 24 h after preparation.

Functional and toxicological studies also suggest a specific ABC distribution pattern along the intestine. For example, in *ex vivo* intestinal preparations from the Patagonian silverside, the apical and basolateral DNP-SG efflux was significantly inhibited by microcystin-LR in the anterior and middle intestine; however, neither apical nor basolateral DNP-SG efflux transport was affected in the posterior intestine (Bieczynski et al., 2016). Table 3 offers a list of representative physiological and toxicological fish ABC proteins’ substrates.

**TABLE 3:** Representative chemical compounds, which interact with fish ABC proteins presented in alphabetical order.

ABC protein	^1^Physiologic, ^2^Drugs/Experimental substrates and ^3^toxicological substrates	References
Abca	^1^Sterols*, phospholipids	Fang et al., 2013, 2014; Kortner et al., 2014*
Abcb4, Abcb5 (Pgp)	^1^Lipophilic compounds, e.g., steroid hormones ^2^Ca-AM, cyclosporin A, daunomycin, doxorubicin*, etoposide, ivermectin, R123, verapamil, vinblastine, and several therapeutic human drugs ^3^Azinphos-methyl, Cd, Hg, MCLR, phenanthrene, Zn	Miller, 1995; Doi et al., 2001; Zaja et al., 2007; Caminada et al., 2008; Zaja et al., 2011; Umans and Taylor, 2012*; Fischer et al., 2013; Lu et al., 2015; Lerebours et al., 2016; Ellis et al., 2018; Gordon et al., 2019; Bieczynski et al., 2021; Robey et al., 2021;
Abcb11 (Bsep)	^1^Bile salts and bile alcohols ^2^DHFDA	Ballatori et al., 2000; Zaja et al., 2008; Ellis et al., 2018
Abcc2 and BL ABCCs (MRPs)	^1^Leukotrienes*, bilirubin, estrogen conjugates, organic anions, other conjugated metabolites, cyclic nucleotides (Abcc4, Abcc5) ^2^Ca-AM*, CDNB*, fluorescein methotrexate*, MK571*, R123, sulforhodamine 101*, ^3^As*, Cd, DDT, Pb, lindane, MCLR*, Hg	Miller et al., 1998*, 2002, 2007; Masereeuw et al., 2000; Reichel et al., 2008; Zaja et al., 2008; Long et al., 2011a,c; Bieczynski et al., 2014, 2016*; Lu et al., 2014, 2021; Painefilú et al., 2019, 2020*
Abcc7 (Cftr)	^1^Chloride* ^3^Mercury*	Ratner et al., 2006*; Weber et al., 2006*; Reviewed by Evans, 2010*; Forrest, 2016*
Abcg2 (Bcrp)	^1^Bile salts, testosterone ^2^Doxorubicin, fenofibrate, furosemide, Ko143, pheophorbide, prazosin, propranolol, sildenafil ^3^BaP, DDE, chlorpyrifos, curcumin (natural product), endosulfan, fenoxycarb, malathion	Zaja et al., 2011, 2016
Abcg5, Abcg8	^1^Neutral sterols* ^3^Lindane	Kortner et al., 2014*; Zhu et al., 2018; Zhang L. et al., 2020

*BaP, benzo(a)pyrene; BL, basolateral; Ca-AM, calcein-AM; CDNB, 1-chloro-2,4-dinitrobenzene; DDE, dichlorodiphenyldichloroethylene; DDT, dichlorodiphenyltrichloroethane; DHFDA, dihydrofluorescein diacetate; MCLR, microcystin-LR; R123, rhodamine 123.*

The asterisk (*) indicates studies performed on fish intestines or rectal glands.

## Toxicological Studies

This section presents the current literature on the effects of environmental pollutants on the expression and function of fish ABC proteins, emphasizing intestinal ones and the available evidence on the role of these proteins in pollutants detoxification. It is important to consider that the primary function of ABC transporters is to pump metabolites and potentially harmful molecules out of the cells. Concerning the individual’s defense, the cellular location of a transporter in a polarized epithelium like the intestinal mucosa, the liver, gills, and kidney tubules determines its function. In the intestinal mucosa, apical transporters, such as Abcb1/4, Abcb5, Abcc2, and Abcg2, play a protective role as a barrier against the absorption of xenobiotics. In turn, basolateral proteins, such as Abcc1, and Abcc3, export toxic compounds to the blood for further detoxification in the liver or the kidney.

### Cyanotoxins

Cyanobacteria are one of the main phytoplankton components of most bodies of water and are thus significant components in the diet of phytoplanktivorous species (Ferrão-Filho et al., 2002; Pires et al., 2004; Xie et al., 2004, 2005). Under favorable conditions, cyanobacteria may reach massive cell densities, called cyanobacterial blooms (see Chorus and Bartram, 1999; Huisman et al., 2018 for reviews). Many species of this group can produce toxic secondary metabolites (cyanotoxins), which affect human and ecosystems health (Wiegand and Pflugmacher, 2005; Huang and Zimba, 2019 for reviews).

Microcystins (MC) are mainly intracellular cyclic peptide toxins produced by cyanobacteria of several genera. Thus, fish’s absorption of these toxins mostly depends on the ingestion and digestion of cyanobacterial cells and occurs in the gastrointestinal tract (Tencalla et al., 1994; Bury et al., 1998; Bieczynski et al., 2013). According to functional and molecular studies on fish, humans, and rodents, this toxin enters the cells through OATP bile acid transporters (Bury et al., 1998; Fischer et al., 2005; Boaru et al., 2006; Feurstein et al., 2010). This toxin exerts its specific effect by inhibiting protein phosphatases 1 (PP1) and 2A (PP2A) by non-covalent and covalent interactions (MacKintosh et al., 1990), disrupting the regulation of many cellular functions and less specific effects like damage to lysosomes and oxidative stress (Carmichael, 1994; Boaru et al., 2006; Amado and Monserrat, 2010 for a review). Inside the cells, a phase II biotransformation reaction catalyzed by enzymes of the family glutathione –S transferase (GST) conjugates MCLR with glutathione (GSH) (Pflugmacher et al., 1998). As analyzed below, the mechanisms for eliminating MCLR are still not entirely established but likely involve ABC protein-mediated transport.

Bieczynski et al. (2014, 2016) have studied the detoxification and efflux mechanisms of MCLR (the most studied and one of the most harmful MC) and other xenobiotics in the anterior, middle, and posterior intestine of the Patagonian silverside and the middle and distal intestine of the rainbow trout using *in vivo*, *ex vivo*, and *in vitro* techniques. Experiments with intestinal non-polarized (strips) and polarized (everted or non-everted sacs) *ex vivo* preparations and combining the studied xenobiotics with specific ABCC substrates, such as the GSH conjugate of 1-chloro-2,4-dinitrobenzene (CDNB), 2,4-dinitrophenyl glutathione (DNP-SG), and calcein; show that MCLR inhibits the efflux activity of apical and basolateral Abcc proteins in both species (Bieczynski et al., 2014, 2016). In addition, the coexposure to MCLR and the specific ABCC inhibitor MK571 increases the toxic effects of MCLR, which indicates that blocking the Abcc transport increases the intracellular MCLR concentration. These results suggest the involvement of apical Abcc2 in MCLR detoxification, while basolaterally located Abcc proteins protect the enterocytes from the remaining toxin.

The *in vivo* exposure of the rainbow trout to MCLR through the diet for 12, 24, and 48 h induces the intestine GST activity. In intestinal strips, the DNP-SG transport rate decreases at 12 h, is recovered at 24 h, and rises at 48 h, suggesting increased Abcc activity. However, this GST and Abcc activity modulation does not correlate with changes in abcc2, gst-π, or gst-ω mRNA expression. The study of polarized preparations shows that the apical DNP-SG transport rate recovers to control levels at 48 h. In contrast, the basolateral transport reaches a significantly higher rate than the control at the same time, suggesting the induction of a basolateral Abcc, such as Abcc1, Abcc3, or Abcc4 (Painefilú, 2021). Kaur et al. (2019) have studied the involvement of human ABC proteins in the excretion of MCs. Using insect cell membrane vesicles overexpressing human ABC transporters together with specific substrates and inhibitors, they have found that only ABCC2 transports MCs in a congener-specific fashion (MCLF > MCLR > MCRR). In contrast, MCs do not interact with ABCB1, ABCB11, ABCC1,3,5, or ABCG2. These results coincide with those described above on the involvement of ABCC2/Abcc2 in MC efflux. However, the substrate affinity of other ABCC-Abcc proteins seems to differ between human and fish transporters and deserves further experiments with more comparable methodologies.

Abcb4 has been related to MCLR transport as well. Lu et al. (2015) showed that MCLR is toxic for zebrafish embryos and causes a concentration-dependent increase in abcb4 transcripts. A whole-mount *in situ* hybridization assay detected this induction in the embryos’ intestinal region. Moreover, mutant embryos (with a non-functional abcb4) suffered higher mortality and accumulated more MCLR than the controls, indicating abcb4 involvement in embryo protection against MCLR.

Paralytic shellfish toxins (PST) are neurotoxic alkaloids produced by marine dinoflagellates and freshwater cyanobacteria that inhibit voltage-gated sodium (and other cations) channels, affecting the membrane potential in nerve and muscle cells (Lagos, 2003; Wiese et al., 2010 for reviews). In mammalian models, intestinal PST absorption proceeds through a paracellular way (Andrinolo et al., 2002a,b; Torres et al., 2007), and they are excreted by glomerular filtration or in the bile (Gessner et al., 1997; García et al., 2004, 2010). Ramos et al. (2018) have reported ABCB1 as a PST transporter.

In contrast, PST transport in the fish intestine or other organs has received little attention. Our laboratory studies show that, at least in part, PST absorption follows a transcellular pathway since PST enter the rainbow trout enterocytes through the luminal membrane and damage the lysosomal membrane. These toxins inhibit basolateral but not apical Abcc-mediated transport. However, the coexposure with the Abcc inhibitor MK571 does not increase the cytotoxic effects of PST, suggesting that these toxins are not Abcc substrates but non-competitive inhibitors (Painefilú et al., 2020).

### Inorganic Pollutants

Glutathione –S transferase -mediated conjugation with GSH is a critical detoxification step for metals and metalloids. These conjugates are then extruded from cells by ABCC transporters (Singhal et al., 1987; Kala et al., 2000; Painefilú et al., 2019).

(Long et al., 2011a,b,c) have analyzed the role of zebrafish Abcc5, Abcc2, and Abcc4 in the detoxification of metals and metalloids, Cd, Hg, Pb, and arsenic (AsIII, AsV). Their qPCR analysis indicates that the exposure of embryos to different concentrations of Cd, Hg, As, or Pb for 24–48 h induces abcc5 mRNA expression. Exposure of adult zebrafish for 24 h induces abcc5 expression in 10 different tissues. In particular, Pb and AsV induce intestine abcc5 transcription, but Cd has the opposite effect. In addition, embryos overexpressing abcc5 have a lower death rate than control embryos upon metal exposure (Long et al., 2011a).

Long et al. (2011c) have studied the RNA expression changes of abcc2 in developing and adult zebrafish exposed to Cd, Hg, AsIII, or Pb. Whole-mount *in situ* hybridization shows that Hg and Pb significantly induce abcc2 expression in the intestine. In contrast, Cd downregulates abcc2 expression in the whole embryo. In accordance, qRT-PCR shows abcc2 induction in adult zebrafish exposed to various concentrations of Hg or Pb for 24 h, particularly in the intestine, liver, and kidney.

In addition, Long et al. (2011b) have developed fibroblast-like ZF4 cells resistant to toxic metals and arsenic (ZF4-Cd) by chronic exposure to Cd and selection. ZF4-Cd cells are more resistant to Cd, Hg, AsIII, and AsV than the wild-type cell line (ZF4-WT). Besides, ZF4-Cd cells show higher GSH content, abcc2, and abcc4 expression, lower expression of abcc5, and a higher Cd elimination rate than ZF4-WT. Coexposure to metals with ABCC or GST inhibitors drastically reduces both cell types’ survival rates.

The results from the three studies by Long et al. (2011a,b,c) indicate the importance of ABCC proteins, GST, and GSH in heavy metals detoxification in different organs, including the intestine in developing and adult zebrafish. Although these studies clearly show that Abcc2 can transport toxic metals and arsenic toward the external medium in the zebrafish intestine, more results about cell location and polarized transport are necessary to understand the role of intestinal Abcc4 and Abcc5 in fish detoxification processes. For example, in humans and rodent models, ABCC4 localizes to the basolateral membrane of enterocytes and hepatocytes while renal tubules present an apical ABCC4 (Van Aubel et al., 2002; De Waart et al., 2012).

In this sense, exposure to Pb induces the mRNA and protein expression of abcc4 in zebrafish tissues (the kidney, gills, and intestine) (Lu et al., 2021). These authors detected a concentration-dependent abcc4 transcripts induction in adult fish exposed to Pb for 24 h, which was highest in the kidney. Besides, abcc4–/– mutant zebrafish suffered higher mortality and accumulated more Pb upon exposure to this metal than WT zebrafish. Furthermore, LLC-PK1 cells transfected with zebrafish abcc4 had a higher ability to detoxify and excrete Pb than the control cells.

A functional experiment with everted and non-everted sacs from rainbow trout middle intestine shows that AsIII enters the enterocytes only from the basolateral side and inhibits the apical DNP-SG transport concentration-dependently. Furthermore, previous *in vivo* exposure to AsIII increases DNP-SG transport rate and GST activity in middle intestine *ex vivo* preparations and induces abcc2 RNA expression in the intestine and liver (Painefilú et al., 2019). In the same work, *ex vivo* intestine preparations from fish previously exposed to AsIII show lower sensitivity to MCLR toxicity than those from control individuals. These results suggest that apical Abcc2 excretes arsenic absorbed either from the intestinal lumen, as AsV and then reduced to AsIII, or from the blood as AsIII. The induction of intestinal Abcc2 enhances the organism’s defense against arsenic, and other Abcc2 substrates, like metals and MCLR.

Besides the critical role of Abccs in metals and metalloids detoxification, some studies relate other ABC proteins to this function, although based only on gene expression data. For example, adult zebrafish exposed to Hg show upregulated intestine abcg2b together with several genes related to xenobiotic biodegradation, development, and oxidative stress (Zhang Q.L. et al., 2020). Abcb10, a mitochondrial ABC protein associated with the cellular response to oxidative stress, is significantly upregulated in muscle tissue of zebrafish exposed to Cu and Cd. Also, abcb10 transcript levels increase in the intestine and other tissues, albeit to a lesser extent (Sabri et al., 2012). Finally, Abcc7 is not a xenobiotic transporter but can be affected by inorganic pollutants. For example, Hg inhibits the Cl transport dose-dependently in the shark rectal gland probably by non-competitive interaction with Abcc7 (Ratner et al., 2006; Weber et al., 2006).

### Pesticides and Pharmaceuticals

Cárcamo et al. (2011) exposed rainbow trout to the antiparasitic compound emamectin benzoate (EMB) and analyzed abcb4, abcc1, and CYP enzymes mRNA expression in tissues through semiquantitative RT-PCR. Their results show upregulation of most of the studied CYP and ABC genes after EMB treatment, with the highest increase in kidney and intestine abcc1 (45- and 97-fold, respectively). The expression of abcb4 was down-regulated in the intestine and slightly affected in other tissues. Although the cited study does not analyze the possible transport of EMB by ABC proteins, it is interesting to notice that, in the intestine, this compound downregulates abcb4 and strongly induces abcc1. This effect would reduce the intestinal xenobiotic excretion capacity and increase the EMB transport into the blood, depending on the affinity of Abcc1 for EMB. The marked abcc1 induction in the kidney suggests a possible increase in the glomerular Abcc1 activity, as reported for streptozocin-treated rats (Quezada et al., 2011), which would facilitate EMB filtration. A recent study by Moraes et al. (2020) shows that glyphosate and a commercial glyphosate formulation (RT) modulate adult zebrafish ABC expression and activity in a tissue-specific fashion. These authors report that 96 h exposure to environmentally relevant glyphosate and RT concentrations (0.1 mg L^–1^) increase the transport activity of the Abcc fluorescent substrate calcein in the intestine and other tissues. This effect is more pronounced in the intestine and gills. In the same study, the expression profiles of five abcc genes differ among tissues. The intestine shows only a modest induction of abcc3 by RT and downregulation of abcc1 by glyphosate and RT. In contrast, the gills show a strong induction of abcc2 expression by RT.

These results that suggest various modulation pathways for Abcc activity in different organs are comparable with those from earlier studies on the arsenic effect on Abcc2 transport activity in killifish (Miller et al., 2007; Shaw et al., 2007). The former authors report that AsIII stimulates Abcc2 transport in killifish renal tubules by a non-genomic mechanism, while the latter find induction of abcc2 by AsIII in the liver of the same species. In this sense, De Anna (2019) reports that chlorpyrifos induces the efflux of the ABCC substrate DNP-SG in rainbow trout intestine *ex vivo* preparations in a concentration-dependent manner. The further study of the polarized transport in everted intestinal sacs exposed to 20 μg L^–1^ chlorpyrifos shows increased DNP-SG transport rate by apical membrane transporters, likely Abcc2, but no change in abcc2 mRNA expression. The fact that chlorpyrifos stimulates DNP-SG transport *ex vivo* instead of producing a competitive inhibition suggests that chlorpyrifos is not an Abcc2 substrate. De Anna et al. (2021) report that the exposure of rainbow trout to chlorpyrifos *in vivo* for 12 h induces the RNA expression of the nuclear receptor pregnane X receptor (PXR) in the intestine and liver, together with an induction of abcc2 only in the intestine but no change in abcb4 expression. In contrast, as described above, AsIII and MCLR are substrates of the rainbow trout intestine Abcc2 and thus competitively inhibit DNP-SG transport *ex vivo*, but only AsIII induces intestinal abcc2 mRNA expression after exposure *in vivo* (Bieczynski et al., 2014, 2016; Painefilú et al., 2019; Painefilú, 2021).

Lu et al. (2014) report that, in 96 hpf zebrafish embryos, abcc4 transcripts appear mainly in the intestine and gills. These authors showed that the overexpression of abcc4 in embryos reduces the mortality rate caused by the organochlorine pesticides DDT and lindane and reduces their accumulation. The expression of a dominant abcc4 mutation has the opposite effects. Accordingly, in the same study, LLC-PK1 cells transfected with the zebrafish abcc4 suffered lower DDT and lindane cytotoxicity than control cells and had higher pesticide transport capacity. Both pesticides also caused abcc4 induction in exposed embryos. The authors propose that Abcc4 is critical for DDT and lindane detoxification.

Zhang L. et al. (2020) reported a dose-dependent induction of abcg5 and abcg8 in zebrafish larvae exposed to lindane (from 96 to 120 hpf). Besides, larvae with Abcg5 or Abcg8 overexpression showed a significantly reduced toxicity and lower lindane accumulation than the control larvae. The co-overexpression of both proteins enhanced their protective effects. These authors conclude that Abcg5/8 transport lindane, in addition to the function of Abcc4 in organochlorine pesticides detoxification reported previously by the same group (Lu et al., 2014). Although the results of this study refer to whole larvae without tissue discrimination, the authors state that abcg5 and abcg8 are mainly present in the intestine and liver. Since in mammalian intestine and liver, ABCG5/8 are apical membrane proteins while Abcc4 locates to the basolateral membrane in both organs (Graf et al., 2003; De Waart et al., 2012), Abcg5/8 seem more suitable to excrete toxic compounds in both organs than Abcc4.

On the other hand, ABCC4 localizes to the apical membrane in the mammalian kidney tubules. Thus, it would export xenobiotics to urine, conferring resistance to the organism. Therefore, understanding the physiological role of ABC proteins in fish requires new studies on their function and cellular location and the regulatory pathways involved in their expression and trafficking to the membrane in different organs.

Love et al. (2021) studied the basal RNA expression of abcb4 and abcb5 in different tissues of the rainbow trout and their inducibility by the antifungal agent clotrimazole. Abcb4 RNA was expressed ubiquitously among tissues, showing its highest expression in the proximal and distal intestine (followed by the liver and several parts of the central nervous system). This transporter showed three orders of magnitude higher expression than abcb5 in the intestine. The intraperitoneal injection of clotrimazole resulted in a significant induction of abcb5 in the optic lobe and the distal intestine (4.4 and 3.2 fold increase, respectively). Clotrimazole is a potent ligand of the human PXR, and there is evidence of PXR activation following exposure to clotrimazole in fish (Kliewer et al., 2002; Moore et al., 2002; Milnes et al., 2008; Bainy et al., 2013). However, few studies exist on the expression of potential PXR-target genes in fish, particularly in the intestine. As detailed above, De Anna et al. (2021) reported the induction of PXR and its target genes abcc2 and cyp2k1 but not abcb4 by chlorpyrifos in the rainbow trout middle intestine, which corresponds to the rear part of the proximal intestine in the Love et al. (2021) study. Both studies coincide in the lack of induction of abcb4 by PXR agonists, such as clotrimazole and chlorpyrifos, in the rainbow trout anterior-mid intestine. In contrast, the abcb5 induction by clotrimazole only in the distal intestine and optic lobe (Love et al., 2021) suggests a tissue-dependent response of this gene. As far as we know, there are no reports on the regulation of abcb5 expression in fish.

### Hydrocarbons

As far as we know, there are no reports on the transport of hydrocarbons or related compounds by ABC proteins in the fish intestine. Bard et al. (2002) have studied the expression of Pgp protein in different tissues of killifish, collected from relatively unpolluted and polluted sites with high levels of polychlorinated biphenyls (PCB) and planar halogenated aromatic hydrocarbons (PHAHs) by immunohistochemistry and immunoblot techniques with the mAb C219. They localized Pgp in epithelial cells of the posterior intestine, with intense staining in more than half of the individuals collected at the polluted site. In contrast, only 3–6% of the fish collected at the control site showed a positive Pgp reaction. In contrast, fish livers from the control site had greater Pgp expression than those from the polluted area.

Costa et al. (2012) characterized the sequences of the ABC genes abcb1-like, abcb11, abcc1, abcc2, abcg2. They analyzed the mRNA expression of those genes together with CYP1A and GSTα in the liver, gills, and proximal intestine of the Nile tilapia exposed to BaP dissolved in water or added to the diet. Control fish showed the highest abcb1-like, abcc2, and abcg2 mRNA relative expression in the anterior intestine, followed by the liver. Among the abcc genes, water-born BaP induced abcc1 and abcc2 in the gills, together with intestine (concentration-dependent) and liver abcg2. Interestingly, dietary BaP only induced gill abcc2, while the solvent added to the diet (acetone) induced intestine abcc1 and abcg2. Both treatments induced CYP1A, a known target of the nuclear receptor AhR, with the highest effect on the intestine. There was no significant effect of BaP in the expression of abcb1-like or abcb11. The results on CYP1A and abcb1-like coincide with a further western blot and immunofluorescence study by the same group (Costa et al., 2013) that shows increased protein expression of CYP1A but not Pgp in Nile tilapia exposed to BaP.

## Discussion and Future Perspectives

Studies on fish ABC proteins show high functional homology with their human counterparts (Luckenbach et al., 2014, for review; Robey et al., 2021). However, the available evidence on their physiological role in the intestine includes only the ABCB, ABCC, and ABCG subfamilies, plus two studies on abca1 and abca2 RNA expression (Kortner et al., 2014; Liu et al., 2016). Toxicological studies show tissue-specific effects (e.g., Bard et al., 2002) and offer clues on the involvement of several regulatory pathways.

Studies on ABCB proteins show high expression of abcb4, especially in the posterior intestine (Lončar et al., 2010; Love et al., 2021). In the rainbow trout, the PXR agonists chlorpyrifos and clotrimazole did not affect intestinal abcb4 expression, while clotrimazole induced abcb5 (De Anna et al., 2021; Love et al., 2021). Among the AhR agonists (related with PCB and hydrocarbons pollution), BaP did not induce abcb1-like or abcb11 in the Nile tilapia (Costa et al., 2012), while in the channel catfish, β-naphthoflavone increased the immunohistochemical label intensity with an anti-Pgp antibody (Kleinow et al., 2000). Since the latter paper and other studies on the effects of hydrocarbons and other inducers of the AhR use anti-mammalian Pgp antibodies that do not discriminate among abcb4, abcb5, and abcb11, the impact of this kind of pollutants on fish intestine ABCB proteins remains unclear.

ABCC transporters are abundant in the fish intestine, but only Abcc2 is present in the apical membrane. Several reports supply evidence related to the excretion of MCLR and arsenic to the intestinal lumen (Bieczynski et al., 2014, 2016; Painefilú et al., 2019) by Abcc2 or the induction of abcc2, abcc4, and abcc5 by the exposure to arsenic and toxic metals associated to increased resistance (Long et al., 2011a,b,c; Painefilú et al., 2019). These results suggest a critical role of intestinal Abcc2 as a barrier against a variety of xenobiotics. While the induction of the basolateral proteins, abcc4 and abcc5 by metals, indicates metals transport to another detoxification organ. Similarly, the increased intestinal and renal abcc1 expression by the pharmaceutical emamectin benzoate (Cárcamo et al., 2011) suggests intestinal drug absorption and renal excretion.

Abcg2, Abcg5, and Abcg8 are present in the fish intestine, being Abcg2 particularly abundant. Kortner et al. (2014) report the expression of abcg5 together with abca1 in the Atlantic salmon pyloric ceca, which suggests the participation of both transporters in the regulation of cholesterol uptake and excretion. Zhang L. et al. (2020) and Zhang Q.L. et al. (2020) reported induction of intestinal abcg5 and abcg8 associated with resistance to the pesticide lindane and upregulation of abcg2b by Hg in zebrafish. Costa et al. (2012) reported the induction of intestinal abcg2 in the Nile tilapia in response to BaP.

Besides the few physiological and toxicological works cited in this review, there is a lack of information about the role of ABC proteins in the physiological functions of the intestine, such as bile compounds secretion and reabsorption and transport of other metabolites. These functions are exciting for the development of new feed ingredients for aquaculture.

Most frequently, polluted environments contain more than one toxic substance or even complex mixes. However, few works deal with the effects of combining xenobiotics on ABC. The studies on the impact of pollutants interaction on these proteins should include traditionally studied ones and others that generate growing concern. For example, microplastics may enter the organism via the gastrointestinal tract and affect ABC-mediated transport. Wu et al. (2019) have reported that polystyrene microplastics inhibit ABC efflux activity in the human colon adenocarcinoma Caco-2 cells and could act as chemosensitizers to other pollutants by increasing their intracellular accumulation.

Another factor to consider in future studies on ABC proteins is the gut microbiome that most likely influences the functions of these transporters. In mammals, the intestinal microbiota metabolizes different drugs and natural metabolites, such as bile salts, also substrates and modulators of ABC proteins. Thus, the intestinal microbiota affects the intestinal absorption and detoxification processes (Klaassen and Cui, 2015, for a review). In fish, there are few studies on microbiome-host toxicokinetic interaction. As far as we know, there are no specific works on the microbiome and ABC proteins interaction. However, xenobiotics, like antibiotics, other drugs, and microplastics, can affect the microbiome composition and metabolism and, therefore, the intestinal function in general and the function of ABC proteins in particular (see Bertotto et al., 2019; Catron et al., 2019 and citations reviewed therein; Ding et al., 2021).

Inflammation and infection processes are almost wholly unexplored concerning ABC protein functions. Mottaz et al. (2017) suggested that bacterial lipopolysaccharides (LPS), which induce an inflammatory response in zebrafish embryos, are eliminated through ABC proteins. These authors exposed embryos to LPS with/without the Abcb4 inhibitor cyclosporine A and found higher LPS accumulation in the gastrointestinal region of the individuals treated with cyclosporine A. Similarly, Neudeck et al. (2004) found that Pgp (expression and function) affects the invasion capacity of the bacteria *Listeria monocytogenes* in the Caco2 cell line and other mammalian models.

Future experiments should consider that a great variety of aquatic pollutants, infectious agents, aquaculture feed components, and therapeutic agents can interact with fish intestinal ABC proteins, affecting their barrier and secretory function. These interactions could be direct or mediated by a change in the microbiome conditions.

## Author Contributions

FB and CL designed and wrote the text. JP performed the graphs. JP and AV collaborated in the revision and editing of the manuscript. All the authors contributed to the article and approved the submitted version.

## Conflict of Interest

The authors declare that the research was conducted in the absence of any commercial or financial relationships that could be construed as a potential conflict of interest.

## Publisher’s Note

All claims expressed in this article are solely those of the authors and do not necessarily represent those of their affiliated organizations, or those of the publisher, the editors and the reviewers. Any product that may be evaluated in this article, or claim that may be made by its manufacturer, is not guaranteed or endorsed by the publisher.
